# Targetless Radar–Camera Extrinsic Parameter Calibration Using Track-to-Track Association

**DOI:** 10.3390/s25030949

**Published:** 2025-02-05

**Authors:** Xinyu Liu, Zhenmiao Deng, Gui Zhang

**Affiliations:** 1 School of Electronics and Communication Engineering, Shenzhen Campus of Sun Yat-sen University, Shenzhen 518107, China; liuxy296@mail2.sysu.edu.cn; 2 School of Electronics and Communication Engineering, Sun Yat-sen University, Guangzhou 510275, China; 3 Dongguan Power Supply Bureau of Guangdong Power Grid Co., Ltd., Dongguan 523008, China; csuzgui@163.com

**Keywords:** targetless calibration, sensor fusion

## Abstract

One of the challenges in calibrating millimeter-wave radar and camera lies in the sparse semantic information of the radar point cloud, making it hard to extract environment features corresponding to the images. To overcome this problem, we propose a track association algorithm for heterogeneous sensors, to achieve targetless calibration between the radar and camera. Our algorithm extracts corresponding points from millimeter-wave radar and image coordinate systems by considering the association of tracks from different sensors, without any explicit target or prior for the extrinsic parameter. Then, perspective-n-point (PnP) and nonlinear optimization algorithms are applied to obtain the extrinsic parameter. In an outdoor experiment, our algorithm achieved a track association accuracy of 96.43% and an average reprojection error of 2.6649 pixels. On the CARRADA dataset, our calibration method yielded a reprojection error of 3.1613 pixels, an average rotation error of 0.8141°, and an average translation error of 0.0754 m. Furthermore, robustness tests demonstrated the effectiveness of our calibration algorithm in the presence of noise.

## 1. Introduction

Sensor fusion means the integration of measurement results from multiple sensors to obtain a more accurate, reliable, and comprehensive understanding of the measured quantities or phenomena. This involves combining measurement results from different sensors, which may differ in their sensing principle, accuracy, precision, and noise characteristics. By fusing measurements from different sensors, the advantages of each sensor can be complemented. In autonomous driving systems, common sensors include radar, lidar, and cameras. While the performance of lidar and cameras significantly deteriorates in rainy and foggy conditions, the performance of millimeter-wave radar remains unaffected. However, millimeter-wave radar lacks precise perception of object texture, due to the sparsity of its data. On the other hand, lidar point clouds and camera images provide rich texture information. Therefore, sensor fusion [1,2,3,4] of radar, camera, and lidar sensors is widely employed in modern robotic systems, such as autonomous vehicles (AV) [5,6,7,8,9], to enhance the system accuracy and robustness. However, the high cost of lidar limits its widespread application, and sensor fusion solutions involving cameras and radar have become a mainstream trend.

Sensor calibration is crucial for sensor fusion, as it plays a vital role in ensuring the accuracy, reliability, and consistency of measurement results from different sensors. Sensor calibration involves determining intrinsic parameters, extrinsic parameters, and time parameters, corresponding to the physical characteristics of each sensor model, the transformation between sensor coordinate systems, and the alignment of sensor clocks, respectively. In multi-sensor systems, manual sensor calibration is a tedious process. Additionally, system re-calibration is required when the relative positions of sensors change. However, most current sensor calibration methods still rely on explicit calibration targets, making the calibration process costly.

Many existing radar and camera calibration methods rely on explicit calibration targets, while some scholars have proposed targetless methods. These methods either require initial extrinsic parameter estimation or rely on specific scenes, such as lanes. Unlike lidar, millimeter-wave radar point clouds are very sparse, so it is difficult to extract significant environmental features from them. Therefore, some targetless calibration methods for lidar and cameras are not suitable for radar and camera calibration. Although there are no identifiable shared environmental features, tracking results for moving objects can be independently obtained from the sensor information of radar and cameras.

**Figure 1:**
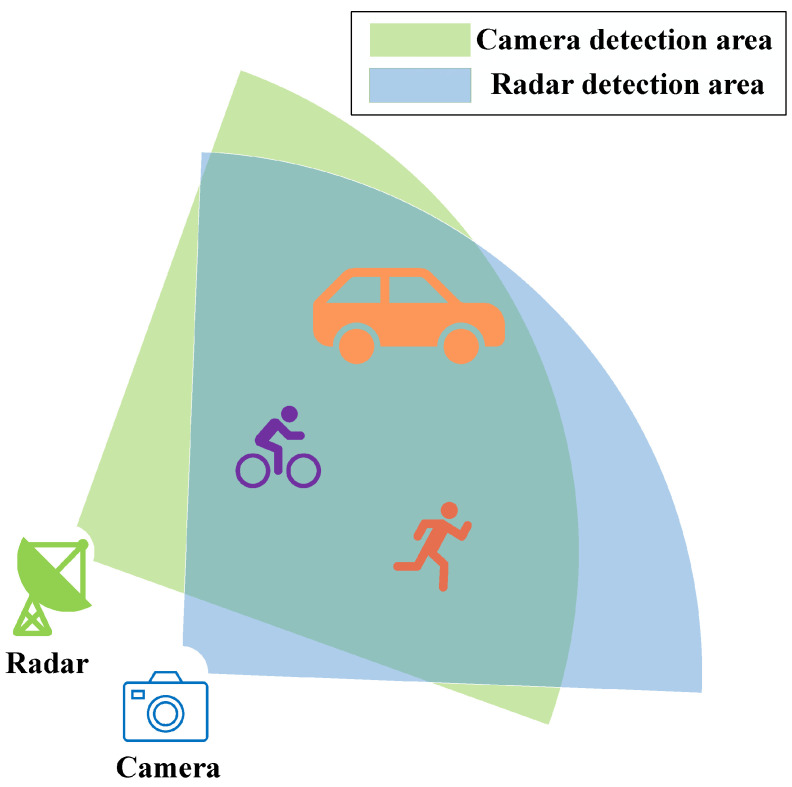
Application scenarios for the algorithm.

To address the issues in current calibration methods, we propose a targetless calibration method that associates the tracking results of the radar and cameras first, and then performs sensor calibration. The study scenario is shown in Figure 1. Our method aims to accurately associate the tracks obtained from each sensor. The algorithm does not require any prior estimation of extrinsic parameters and obtains corresponding 2D–3D point correspondences. After obtaining the 2D–3D correspondences, we use the perspective-n-point (PnP) algorithm to compute the initial values of the extrinsic parameters and improve the accuracy of the calibration results using a nonlinear optimization algorithm. In an outdoor experiment, our algorithm achieved a track association accuracy of 96.43% and an average reprojection error of 2.6649 pixels. On the CARRADA dataset, our calibration method yielded a reprojection error of 3.1613 pixels, an average rotation error of 0.8141°, and an average translation error of 0.0754 m. Furthermore, robustness tests demonstrated the effectiveness of our calibration algorithm in the presence of noise.

Our initial idea was that each track pair could yield a set of extrinsic parameters. Consequently, we could assess the quality of track association and obtain correct track pairs using these extrinsic parameters. Thus, prior knowledge of the extrinsic parameters is not necessary during this process. Well-matched track pairs provide abundant corresponding points, which is crucial for obtaining accurate extrinsic parameters. The main contributions of this paper are as follows:

1. We propose a track association algorithm for calibration results that does not require prior knowledge and demonstrated its accuracy in multiple scenarios. The proposed method aims to extract target features from the temporal dimension.
2. Based on the proposed track association and convex optimization algorithm, we achieved high-precision extrinsic parameter calibration of radar and camera without any explicit target or prior for extrinsic parameters.
3. The proposed calibration algorithm is applicable to various scenarios, including autonomous driving and surveillance safety. The calibration algorithm does not require high-quality tracking and allows for tracking errors.

## 2. Related Work

### 2.1. Target-Based Calibration

Many existing calibration methods use manually designed markers to obtain corresponding points from the coordinate systems of radar and cameras. Domhof et al. [10] proposed a single calibration target design and implemented their approach in an open-source tool with connections to the Robot Operating System (ROS). Kim et al. [11] proposed a calibration method between 2D radar and cameras using point matching. They used a corner reflector calibration target that focused the radar signal at the center of the target. Cheng et al. [12] proposed a flexible method for extrinsic calibration of 3D radar and cameras. The proposed method does not require a specially designed calibration environment. Instead, it places a single corner reflector (CR) on the ground to collect radar and camera data simultaneously using the Robot Operating System (ROS). It obtains radar–camera point correspondences based on their timestamps, uses these point correspondences as input to solve the perspective-n-point (PnP) problem, and finally obtains an extrinsic calibration matrix. Agrawal et al. [13] introduced a new method for auto-calibrating 3D radar, 3D lidar, and red-green-blue (RGB) mono-camera sensors using a static multi-target-based system. The proposed method can be used with sensors operating at different frame rates without time synchronization. Song et al. [14] proposed a new method of spatial calibration between a mono camera and 2D radar. Using an augmented reality (AR) marker designed to be detectable by radar, they simultaneously measured the position of the marker concerning the coordinate system of the camera and radar. These methods can obtain accurate extrinsic parameters using precise marker points, but they rely on human participation, and the markers themselves also introduce additional costs to the system.

### 2.2. Targetless Calibration

Some calibration methods do not rely on artificial markers, but these are limited to road traffic. Du et al. [15] proposed a novel spatio-temporal synchronization method of asynchronous roadside MMW radar and camera for sensor fusion. Based on the consistent time flow rate of separate sensors, multiple virtual detection lines were set up to match the time headway of successive vehicles and conduct objective matching to track data. A synchronization optimization model was formulated, and a constrained nonlinear minimization solver was applied to tune the parameters. Liu et al. [16] introduced an intelligent method to calibrate radar and camera sensors for data fusion. They collected information from radar and cameras on the road, with only one target to obtain corresponding points. Then, they used a neural network to learn the mapping from the image coordinate system to the radar coordinate system. Scholler et al. [17] proposed a data-driven method for automatic rotational calibration without dedicated calibration targets. The proposed method ignores the translation between the radar and camera during calibration and requires initial extrinsic parameter estimation. Some methods use the motion of the sensor platform itself to calibrate 3D radar and cameras. Wise et al. [18] presented a continuous-time 3D radar-to-camera extrinsic calibration algorithm, which required manual association of radar and camera detection. J. Peršić et al. [19] proposed a multi-sensor calibration method based on dynamic target tracking, but the proposed association algorithm is relatively simple and may not be suitable for more complex target trajectories. Cheng et al. [20] performed deep learning to extract common feature from raw radar data and camera features. However, due to the significant number of outliers, the accuracy of the calibration results was not satisfactory.

### 2.3. Track-to-Track Association

Track-to-track association refers to finding multiple tracks for the same target using different sensor systems. Existing track association algorithms [21,22] rely on transforming tracks from different sensors to the same coordinate system. In most spatial registration algorithms, data association problems are assumed to have been solved. Similarly, in data association algorithms, spatial registration should be completed. In practice, data association and spatial registration are usually coupled. Shastri et al. [23] and Li et al. [24] achieved targetless calibration within a radar network by correlating radar target tracking results from multiple radars targeting the same object. However, these algorithms primarily focus on track association of the same type of sensors, with limited research on track association between different types of sensors.

## 3. Problem Statement

Extrinsic parameter calibration refers to estimating the rigid transformation between the coordinate systems of two sensors. The rigid transformation matrix from the camera coordinate system to the radar coordinate system is defined as T, which consists of a 3 × 3 rotation matrix R and a 3D translation vector t, and can be represented in the following form:

(1) $$T=\left[\begin{array}{cc} R & t\\ 0_{3}^{T} & 1 \end{array}\right]$$

The coordinates in the radar coordinate system are defined as $\left(x_{r},y_{r},z_{r}\right)$, the coordinates in the camera coordinate system as $\left(x_{c},y_{c},z_{c}\right)$, and the coordinates in the image pixel coordinate system as (u,v). The following relationship is then established:

(2) $$\left[\begin{array}{c} x_{c}\\ y_{c}\\ z_{c}\\ 1 \end{array}\right]=\left[\begin{array}{cc} R & t\\ 0_{3}^{T} & 1 \end{array}\right]\left[\begin{array}{c} x_{r}\\ y_{r}\\ z_{r}\\ 1 \end{array}\right]$$

When camera distortion is not considered, the relationship between image pixel coordinates and camera coordinate system coordinates can be expressed as

(3) $$\left[\begin{array}{c} u\\ v\\ 1 \end{array}\right]=sK\left[\begin{array}{c} x_{c}\\ y_{c}\\ z_{c} \end{array}\right]$$

where K is the intrinsic matrix of the camera and *s* is the scale factor. In this paper, it is assumed that the intrinsic parameters of the camera have been calibrated, and only the calibration of the extrinsic parameters is considered.

**Figure 2:**
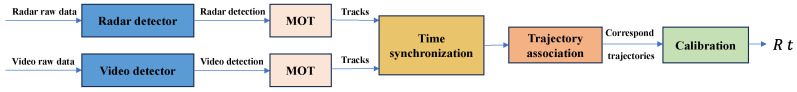
Flowchart of the proposed algorithm. The radar detector includes components such as moving target indication (MTI), constant false alarm rate (CFAR), and fast Fourier transform (FFT). The video detector utilizes YOLOv5 and filters out targets, except for vehicles and people. The MOT refers to multiple object tracking. Through object detection and tracking, target tracks are obtained from raw data. After time synchronization and track association, we can obtain track pairs in the radar and pixel coordinate system. Finally, the extrinsic parameters are obtained using PnP and nonlinear optimization algorithms.

Targetless calibration refers to estimating the extrinsic parameters of the radar coordinate system to the camera coordinate system, without relying on designed markers or explicit calibration targets. When estimating extrinsic parameters, our algorithm only uses information obtained by the sensors. The task is to estimate extrinsic parameters by using unordered point clouds and images provided by radar, without directly providing matching 2D–3D points, which is also known as the blind PnP [25] problem. The entire calibration algorithm flow is shown in Figure 2.

## 4. Proposed Algorithm

In the proposed algorithm, object detection and tracking are first performed on the original image and radar data to obtain the target track. Before track association, time synchronization is achieved according to the sampling rates of the different sensors. Then, the correct track pairs are obtained, and calibration is performed after a certain number of 2D–3D point pairs have been obtained. It is noted that the radar signal processing part of the algorithm was only used in the outdoor experiments. The algorithm was validated on the public dataset CARRADA [26] to ensure its accuracy.

### 4.1. Radar Signal Preprocessing

**Figure 3:**
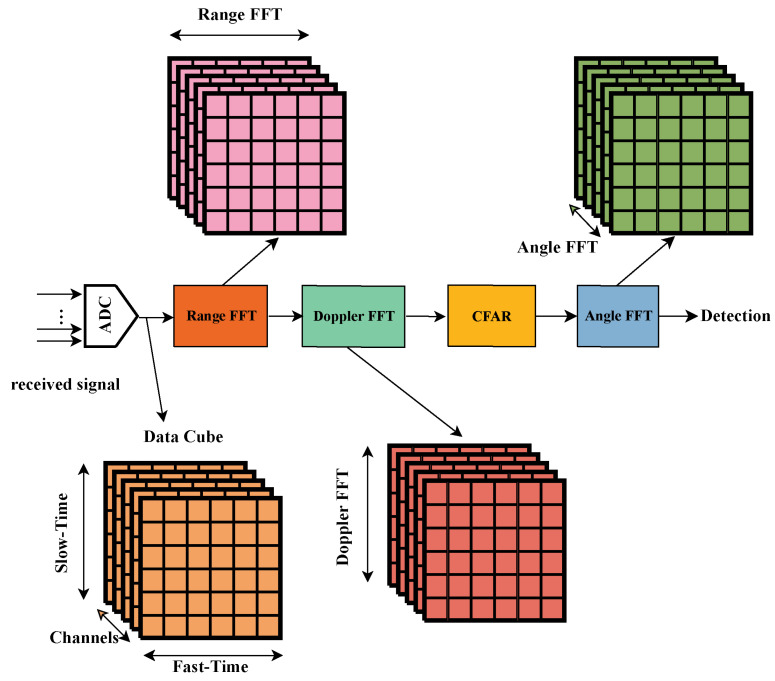
Radar signal processing pipeline.

**Figure 4:**
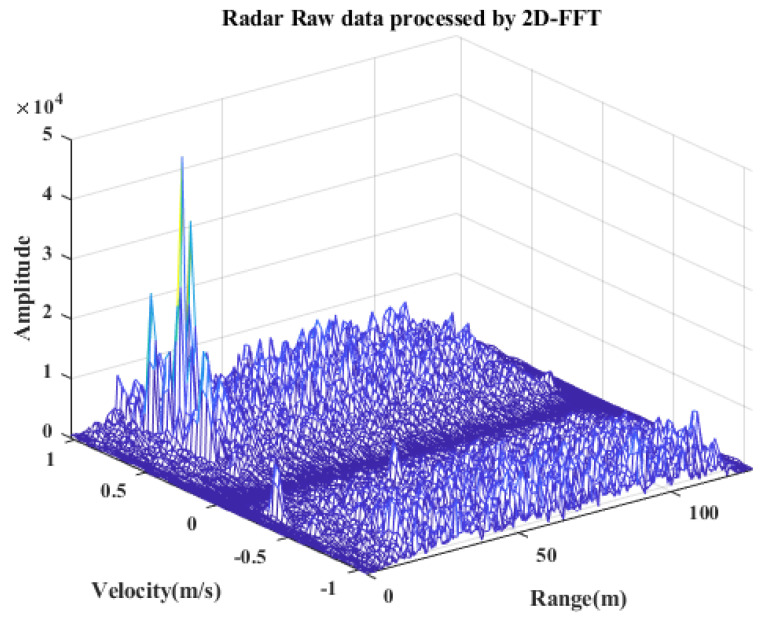
Radar data after MTI and 2D-FFT. The data were collected from the real world. In this scenario, there are three individuals moving, corresponding to the three marked peaks.

The basic radar signal processing pipeline is shown in the Figure 3. First, the received signal is sampled by the analog-to-digital converter (ADC). The moving target indication (MTI) algorithm is applied to the raw radar data for the purpose of static clutter suppression. The fast Fourier transform (FFT) in the range dimension is applied to each sampled intermediate frequency signal for all frequency-modulated frames and virtual receive channels. Then, the FFT in the Doppler dimension is performed along each row of each chirp to obtain a range–Doppler map of each virtual receive channel, which is shown in Figure 4. Cell average constant false alarm rate (CA-CFAR) detectors are applied to detect targets in the range–Doppler map for a balance of speed and accuracy. After CFAR processing, the angle FFT algorithm is applied to obtain the angles of the targets.

### 4.2. Point Cloud Clustering

The mmWave radar data in each frame are a set of points, where each point is represented by a 3D vector composed of coordinates on *x* (left to right), *y* (back to forth), and the radial velocity (velocity along the *y*-axis) $v_{i}$. We denote the *i*-th point by

(4) $$p_{i}:=\left(x_{i},y_{i},v_{i}\right)\in R^{3}$$

The radar echoes contain clutter and noise, which can lead to false positive points in the detection results. Moreover, multipath reflection generates multiple detections from the same target. Density-based spatial clustering of applications with noise (DBSCAN) [27] is used. DBSCAN defines a cluster based on density to identify foreground objects that can be grouped as clusters, while those from unwanted noise are usually scattered in low density. This is primarily utilized for discovering clusters of arbitrary shapes and identifying noise data, so it does not require specifying the number of clusters and is very suitable for unknown target numbers. When DBSCAN is performed, the distance between two points is defined as follows:

(5) $$d^{2}\left(i,j\right)={\left(x_{i}-x_{j}\right)}^{2}+{\left(y_{i}-y_{j}\right)}^{2}$$

### 4.3. Point Target Tracking

A lot of work has been carried out on people tracking based on millimeter wave data. Based on the works of Shuai et al. [28] and Zhao et al. [29], a simple tracking module was constructed. Through tracking the target, false positive points in the detection can be further eliminated. To associate targets from different frames, the Hungarian algorithm [30] is used, and the Euclidean distance between the target centers is selected as the matching metric. $w_{i,N}$ is defined as the observed value of the center of the *i*-th target in the *N*-th frame.

(6) $$w_{i,N}:=\left(x,y,v_{y}\right)\in R^{3}$$

where $v_{y}$ is the velocity on the *y*-axis. Since the center of the cluster is not always accurately located at the target center, there may be a large deviation in the target center from adjacent frames. The Kalman filter is used to smooth the position of the target. Taking a 2D radar as an example, in frame *N*-1, the state vector of the Kalman filter is

(7) $$s_{i,N-1}:=\left[x,y,v_{x},v_{y}\right]\in R^{4}$$

where $v_{x}$ is the velocity on the *x*-axis. In frame *N*, the uniform motion model is selected to predict the new state vector $s_{i,N-1}^{\prime }$ of the target center. Then, the state vector $s_{i,N-1}^{\prime }$ is corrected to $s_{i,N-1}$ by the Kalman filter according to the observed value $w_{i,N}$, as shown in the following formula:

(8) $$s_{i,N}=s_{i,N}^{\prime }+G\left(w_{i,N}-Hs_{i,N}^{\prime }\right)$$

where $G\in R^{3\times 4}$ is the Kalman gain matrix and $H\in R^{3\times 4}$ is the observation model matrix.

**Figure 5:**
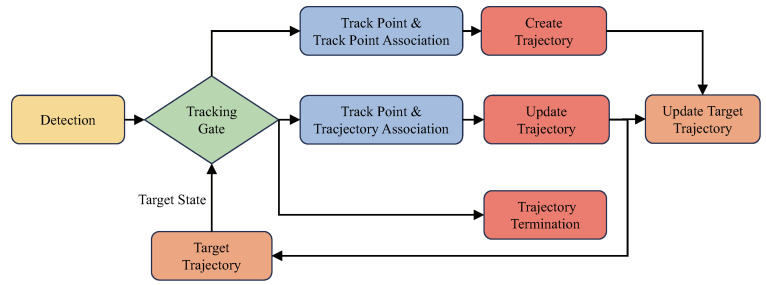
Flowchart of radar target tracking.

**Figure 6:**
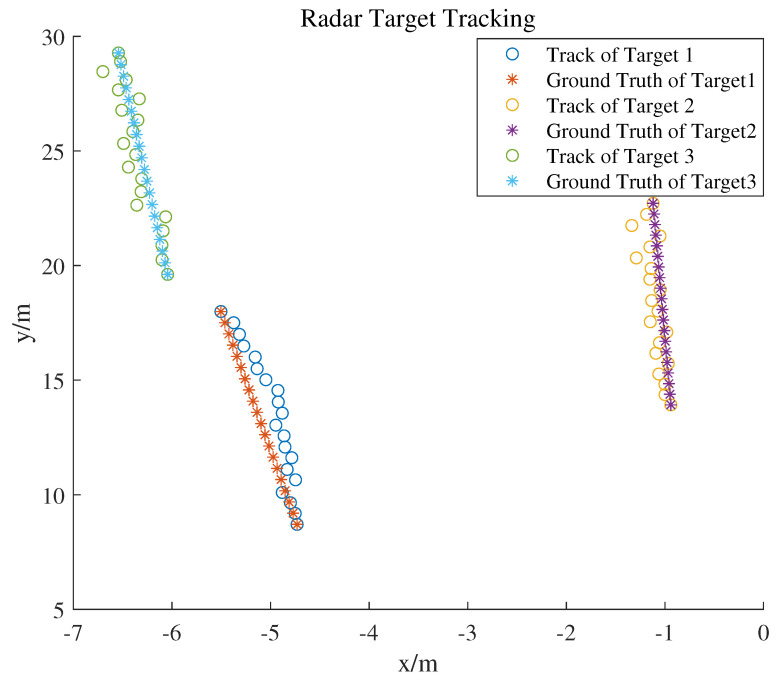
Result of radar target tracking.

A flowchart of radar target tracking is shown in Figure 5. Figure 6 is a radar target track diagram. During tracking, track points are initially preprocessed using DBSCAN. Clusters of track points are then subjected to tracking gate association with existing target tracks, which can result in three scenarios:

1. Track points fall within the tracking gate of an existing trajectory, indicating candidate points associated with tracks, and the most suitable track point–trajectory pair is selected for state update of the trajectory using a Kalman filter.
2. The tracking gate of a track contains no points, indicating either no target was detected by the radar in that frame or the target has disappeared from the radar observation area. If no targets are detected over a period, the target is considered disappeared and the corresponding trajectory is terminated.
3. Track points that do not fall within the tracking gate of any trajectory are associated with other track points. This association indicates the appearance of a new target, prompting the creation of a new trajectory.

Acquiring the ground truth for radar tracking trajectories in real-world settings is inherently difficult. The ground truth is often derived from more accurate observations, such as those from cameras or lidar devices. To assess the radar’s tracking accuracy, experiments were conducted where individuals moved at a uniform speed along predetermined straight lines. The linearly interpolated trajectories of these individuals served as the reference ground truth for our analysis. A detailed performance evaluation is presented in Table 1. The evaluation was performed using an Intel i7-11700F CPU. MAE stands for mean absolute error.

### 4.4. Video Object Detection and Tracking

The YOLOv5 model (https://github.com/ultralytics/yolov5, accessed on 15 November 2023 ), a one-stage object detection framework, was employed to achieve object detection from images. Region proposals are not required by YOLOv5, and features are directly extracted from images to predict the position and category of objects. To eliminate interference from other objects, detection was only performed on humans, bicycles, and vehicles.

**Figure 7:**
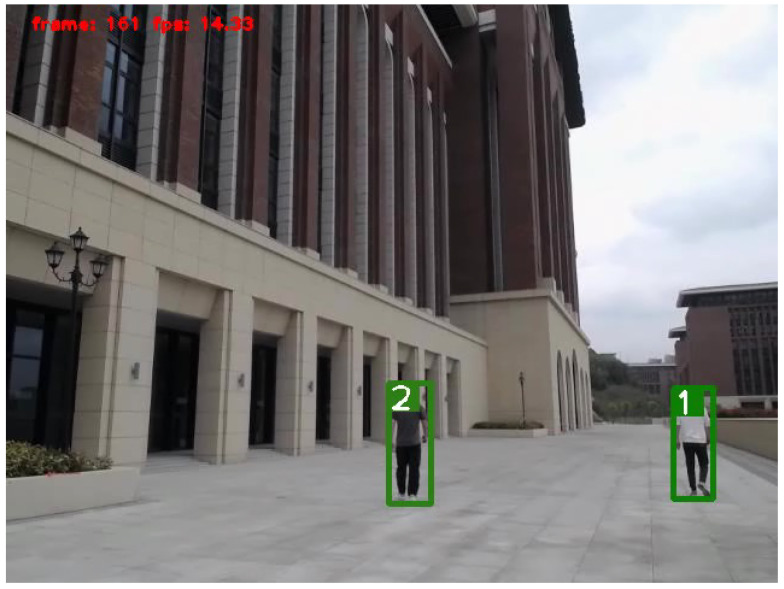
Tracking scenario with multiple pedestrian targets being tracked.

To track multiple objects in videos, the simple online and real-time tracking (SORT) [31] algorithm was utilized to track the detected objects. Similarly to radar-based multi-object tracking, SORT employs Kalman filters to predict the state of objects and utilizes the Hungarian algorithm to associate objects across different frames. The results of the radar-based object tracking and video-based object tracking are shown in Figure 6 and Figure 7. In the provided scenario, video-based object tracking demonstrated a higher accuracy. However, the results of the radar-based object tracking still included some objects (apart from humans and vehicles) that were not interesting to us.

### 4.5. Temporal Synchronization

In order to synchronize the data frames from radar and camera sensors, it is necessary to account for the difference in their sampling rates. Taking radar and camera sensors as examples, the initial sampling moments of the two sensors are different. After time synchronization, the sampling moments are aligned. Let $x_{1}$ represent the measurement value obtained by the radar sensor at time $t_{1}$, $x_{2}$ represent the measurement value obtained by the camera sensor at time $t_{2}$, and $x_{3}$ represent the measurement value obtained by the camera sensor at time $t_{3}$. Let $t_{2}<t_{1}<t_{3}$, and the measurement value of sensor B at a certain time can be obtained by interpolating $x_{2}$ and $x_{3}$:

(9) $$x=x_{2}+\frac{t_{1}-t_{2}}{t_{3}-t_{2}}\left(x_{3}-x_{2}\right)$$

**Figure 8:**
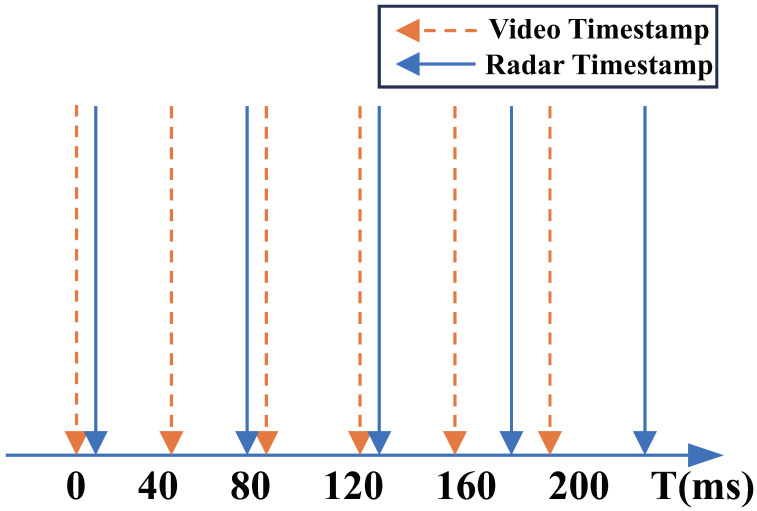
Schematic diagram of temporal synchronization, where solid lines represent radar data frames and dashed lines represent video data frames.

The timestamps of the radar and camera are illustrated in Figure 8, where solid lines represent video data frames and dashed lines represent radar data frames.

### 4.6. Track Association

**Figure 9:**
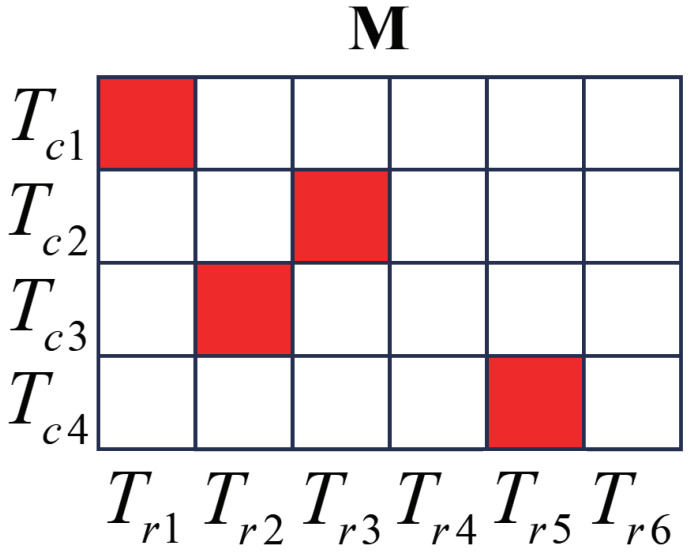
Illustration of the cost matrix M. The red cells represent the minimum value in each row of the matrix, which also corresponds to the radar track with the minimum association cost for each video track. The correct track pairs are $\left(T_{c1},T_{r1}\right)$, $\left(T_{c2},T_{r3}\right)$, $\left(T_{c3},T_{r2}\right)$, $\left(T_{c4},T_{r5}\right)$.

Most existing track association methods are designed for the same type of sensor tracks. Other track association algorithms for heterogeneous sensors are based on transforming the target tracks obtained from different sensors to a common coordinate system. A method for associating the tracks of the same target from radar and video was developed by considering the similarity between tracks from heterogeneous sensors. Figure 9 shows a flowchart of track association. The following algorithm was proposed.

Assume there are *m* tracks $\left\{T_{c1},T_{c2},\ldots ,T_{cm}\right\}$ in the pixel coordinate system and *n* tracks $\left\{T_{r1},T_{r2},\ldots ,T_{rn}\right\}$ in the radar coordinate system. The key to targetless calibration is to obtain corresponding points in the different coordinate systems. The track pair of the same target should be correctly associated, which is essentially a two-dimensional assignment problem. An *m* × *n* matrix M is used to quantify the matching cost between tracks from the radar and camera. Let $R_{i,j}$ and $t_{i,j}$ represent the rotation matrix and the translation vector calculated by $T_{ci}$ and $T_{rj}$. $L_{\mathrm{self}}\left(i,j\right)$ denotes the self-calibration error of $T_{ci}$ and $T_{rj}$, while $\varepsilon \left(l,i,j\right)$ denotes the reprojection error in *l*-th frame. The N(i,j) represents the number of frames overlapping in time between $T_{ci}$ and $T_{rj}$. Let π(·) represent the mapping from the camera coordinate system to the pixel coordinate system, as shown in (3), which is only determined by the intrinsic parameters of the camera. Then, $L_{\mathrm{self}}\left(i,j\right)$ can be calculated through the following formula:

(10) $$\varepsilon \left(l,i,j\right)={∥\pi \left(R_{i,j}p_{j,l}+t_{i,j}\right)-q_{i,l}∥}_{2}^{2}$$

(11) $$L_{self}\left(i,j\right)=\frac{1}{N(i,j)}\sum_{l=1}^{N(i,j)}\varepsilon \left(l,i,j\right)$$

where $p_{i,l}$ represents the *l*-th radar coordinate in $T_{ci}$, while $q_{i,l}$ represents the *l*-th pixel coordinate in $T_{rj}$. An obvious fact is that if $T_{ci}$ and $T_{rj}$ come from the same target, $L_{\mathrm{self}}\left(i,j\right)$ will exhibit a lower level of correlation. However, relying solely on self-calibration errors does not guarantee accurate association. If there are multiple radar tracks with similar shapes (such as walking tracks of multiple people), they will also be similar after certain a rotation and translation. That means the self-calibration error of these radar tracks and $T_{ci}$ will also exhibit a lower level of correlation.

To select the correct track pairs from the similar tracks, for a selected track pair $\left(T_{ci},T_{rj}\right)$, the extrinsic parameter will be validated on the video target track outside of $T_{ci}$ after calculating the corresponding $R_{i,j}$ and $t_{i,j}$. $L_{\mathrm{valid}}\left(i,j\right)$ is used to represent the validation error of this track pair. $T_{ci}^{\prime }$ represents the video target track used for validation. The selection criterion for the track is as follows:

(12) $$T_{ci}^{\prime }=\left\{\begin{array}{cc} T_{c1} & \;\mathrm{if}\;i\neq 1\\ T_{c2} & \;\mathrm{if}\;i=1 \end{array}\right.$$

Let $\left(T_{ci},T_{rj}\right)$ represent the correct track pair; then, the extrinsic parameters calculated from the pair are more accurate. When these extrinsic parameters are used to project all radar tracks onto the pixel coordinate system, a track projection similar to $T_{ci}^{\prime }$ will be generated. The form of $L_{\mathrm{valid}}\left(i,j\right)$ is as follows:

(13) $$L_{\mathrm{valid}}\left(i,j\right)=\min_{k=1,\ldots ,n}\left\{\frac{1}{N(i,j)}\sum_{l=1}^{N(i,j)}\varepsilon \left(l,i,j\right)\right\}$$

In track association, $L_{\mathrm{self}}$ and $L_{\mathrm{valid}}$ are considered to be equally important, and the association cost of $\left(T_{ci},T_{rj}\right)$ can be represented by M(i,j):

(14) $$M\left(i,j\right)=L_{\mathrm{self}}\left(i,j\right)+L_{\mathrm{valid}}\left(i,j\right)$$

### 4.7. Acquiring Radar–Camera Corresponding Points

For each $T_{ci}$, the radar target track $T_{rj}$ is associated with the minimum association cost as its matching track, denoted as $j=\underset{j=1,\ldots ,n}{\arg\min}M\left(i,j\right)$. In practice, the number of radar targets and video targets are generally unequal. To deal with conflicting associations, the track pair with the smaller association cost is trusted. The track pair $\left(T_{ci},T_{rj}\right)$ that does not have a temporal overlap is considered unassociated. To ensure the accuracy of the calibration, a threshold is set on the validation error to avoid track pairs with large measurement error.

### 4.8. Calibration

Once the track pairs from the radar and camera have been correctly associated, the corresponding point pairs in the radar and pixel coordinate systems are obtained. ${\left[x_{r},y_{r},z_{r},1\right]}^{T}$ is used to represent the homogeneous coordinates of the target in the radar coordinate system, and ${\left[x_{\mathrm{nor}},y_{\mathrm{nor}},1\right]}^{T}$ is used to represent the normalized homogeneous coordinates in the camera coordinate system. The relationship between the two is as follows:

(15) $$z_{\mathrm{nor}}\left(\begin{array}{c} x_{\mathrm{nor}}\\ y_{\mathrm{nor}}\\ 1 \end{array}\right)=\left[\begin{array}{cc} R & t \end{array}\right]\left(\begin{array}{c} x_{r}\\ y_{r}\\ z_{r}\\ 1 \end{array}\right)$$

${\left[u,v,1\right]}^{T}$ is used to represent the homogeneous coordinates of the target in the pixel plane. The homogeneous coordinates of the camera plane are denoted as ${\left[x_{c},y_{c},1\right]}^{T}$. The relationship between the two is as follows:

(16) $$\left(\begin{array}{c} x_{c}\\ y_{c}\\ 1 \end{array}\right)=K^{-1}\left(\begin{array}{c} u\\ v\\ 1 \end{array}\right)$$

The estimated extrinsic parameters are assumed to be $\vartheta =\left\{\alpha ,\beta ,\gamma ,T_{x},T_{y},T_{z}\right\}$, and the aim is to minimize the error of the normalized coordinates of the corresponding points in the camera coordinate system. Therefore, the estimated value $\vartheta_{\mathrm{opt}}$ of ϑ is obtained by optimizing the following objective function:

(17) $$\vartheta_{\mathrm{opt}}=\underset{\vartheta }{\arg\min}{∥x_{\mathrm{nor}}-x_{c}∥}_{2}^{2}+{∥y_{\mathrm{nor}}-y_{c}∥}_{2}^{2}$$

During calibration, the height of the target contact point with the ground is set as $z_{r}=0$, denoted as point A, and the center point B at the bottom of the target in the image coordinate system is selected as the match for point A. The extrinsic parameters and reprojection error are then computed. For visualization of the calibration results, the target height is set to $z_{r}=1.0$ m, resulting in projections closer to the target center.

## 5. Experimental Results

### 5.1. Outdoor Experiment

**Figure 10:**
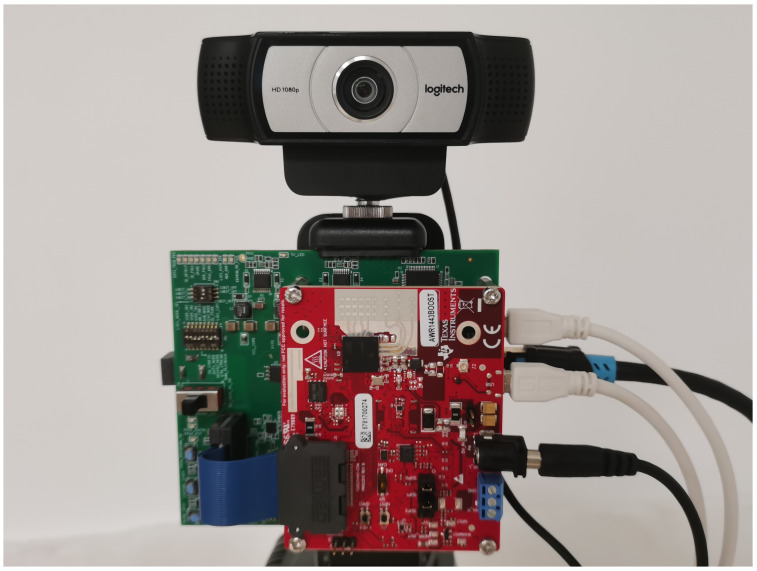
Experimental setup.

To validate the accuracy of the proposed algorithm, validation was first conducted on an outdoor dataset. The data collection scenario was set in a playground with multiple pedestrians walking. A TI AWR1443BOOST millimeter-wave radar and a camera with a resolution of 640 (width) × 480 (height) pixels were utilized. The systematic errors [32] of both the radar and camera were accounted for and calibrated in advance. The experimental setup is shown in Figure 10. The camera was placed above the radar, and they were both securely fixed on a mount. The parameters of the AWR1443BOOST are shown in Table 2.

A Matlab workstation was used to control the radar and camera for simultaneous data acquisition. A total of 30 sets of radar data, with the number of people in the scene ranging from 2 to 6, were collected. Ten sets of data were selected as the validation set, and the calibration results obtained from the remaining 20 sets of data were analyzed. The calculation method for the reprojection error (RE) was defined as follows:

(18) $$\mathrm{RE}=\frac{\sum_{i=1}^{N_{T}}\sum_{j=1}^{N_{f}}\sqrt{{\left(u_{ij}-u_{ij}^{\prime }\right)}^{2}+{\left(v_{ij}-v_{ij}^{\prime }\right)}^{2}}}{N_{f}*N_{T}}$$

where $N_{f}$ and $N_{T}$ represent the number of frames in a track pair and the number of targets. MA represent the mean accuracy of the track association. $u_{ij}$ and $v_{ij}$ denote the pixel coordinate in the *j*-th frame of the *i*-th target, while $u_{ij}^{\prime }$ and $v_{ij}^{\prime }$ represent the coordinate in the *j*-th frame of the *i*-th target projected onto the pixel coordinate system from the radar coordinate system.

Data association methods from [19,20] were evaluated. Specifically, the following comparison experiments were designed. It is important to note that the focus was placed solely on the differences in data association methods, while consistency was maintained by using the same optimizer during calibration.

1. Approach A: The data association method from [19], which considers only the average velocity norm and average position norm of tracks during data association, was applied. To adapt this algorithm to the dataset, irrelevant radar target tracks were manually filtered out before applying the association method, thus eliminating the need to resolve conflicts in track association.
2. Approach B: In [20], a target discriminator was constructed to determine whether the radar targets and image targets corresponded. Unfortunately, the specific discriminator used in this project is not publicly available. During the experiment, the discriminator was simulated and the same discrimination accuracy as reported by the original authors was maintained. For targets that were incorrectly distinguished, they were randomly assigned to another target.
3. Approach C: In [33], a novel target-based calibration method was proposed. The experimental results from the original text were included for comparison.
4. Approach D: A target-based [11] calibration method.
5. Approach E: A target-based [12] calibration method.
6. Single target calibration: Scenarios with a single target were simulated by selecting the most likely track pair to obtain corresponding points, rather than using all possible correspondences.

The experimental results are given in the Table 3, with an average RE of 2.6649 pixels for multiple-track pairs and 5.9168 pixels for single-track pairs. Compared to the other data association schemes, the scheme using the multiple targets for calibration achieved a smaller RE, while the RE of approach A and B were 31.53 pixels and 11.998 pixels. The accuracy of the proposed algorithm far exceeded that of certain existing target-based methods. Although there was a small performance gap compared to the existing state-of-the-art target-based methods, the method proposed in this paper does not require calibration boards or manual selection of radar–image point pairs, making it more flexible and cost-effective.

**Figure 11:**
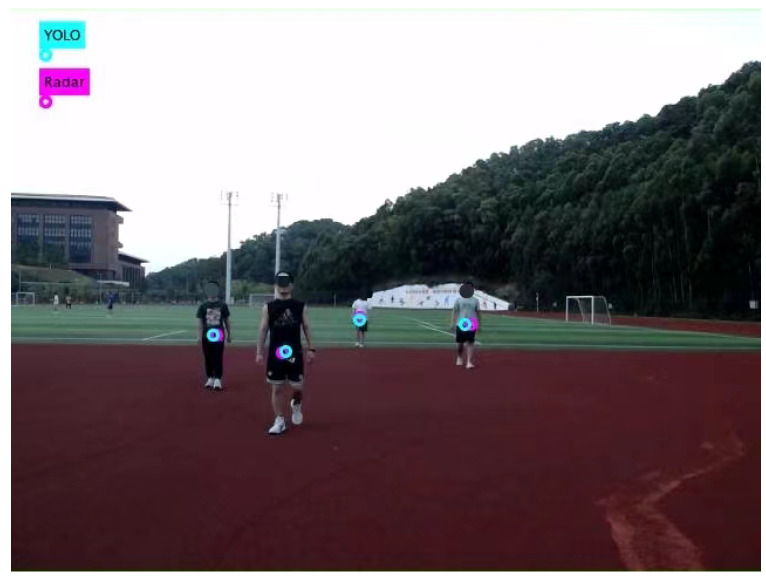
Projection of radar points.

The impact of different track association metrics on the association accuracy was also analyzed, as shown in Table 4. The accuracy of $L_{\mathrm{self}}$ was found to be 71.34%, while the accuracy of $L_{\mathrm{valid}}$ was 64.29%. Using $L_{\mathrm{self}}$ and $L_{\mathrm{valid}}$ as the track association metrics was found to be insufficient for meeting the required precision, while the combined metric denoted as M achieved an accuracy of 96.43%. Approach A was found to be more suitable for simple traffic scenarios. However, in the outdoor experiments, an accuracy of only 35.7% was achieved. For Approaches B and C, the association accuracy could not be provided as it had not been truly employed for track association. Figure 11 shows the projection of the radar object detection onto the image, which was near the target center given by YOLOv5.

Table 5 shows the time consumption of the various methods. Although the proposed method involved an additional mn iterations for parameter optimization compared to the comparison methods, the conventional steps of radar signal processing took up a significant portion of the time. Considering the performance improvement, this extra computation time is acceptable. In addition to the time spent on signal processing, method C also consumed time in manually placing the markers and selecting point pairs, which generally required more time.

### 5.2. CARRADA Dataset

Since the targets in the outdoor experiment were humans, the aim was to extend the target category to vehicle targets. The CARRADA dataset provides tracking results and accurate extrinsic parameters, which are given by manual calibration. The dataset is available at https://github.com/valeoai/carrada_dataset, accessed on 1 December 2023. It enabled us to apply our track association and calibration algorithm.

**Figure 12:**
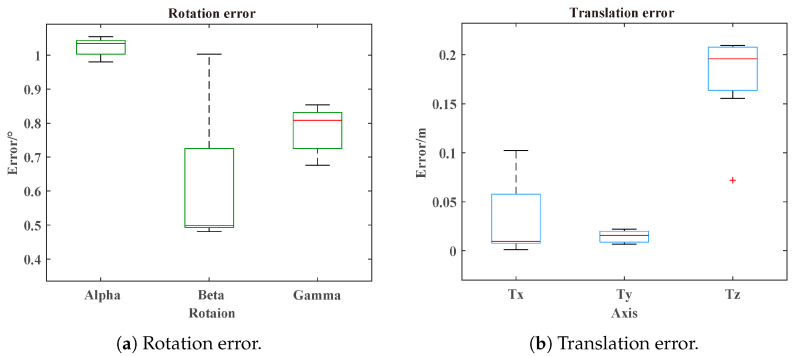
Rotation and translation error in the CARRADA dataset. The ends of the box in the figure represent the upper and lower quartiles, respectively, while the red line denotes the median. The plus signs indicate the outliers. The ends of the box, extended by dashed lines, represent the maximum and minimum values within the normal range.

The tracking results provided by the CARRADA dataset were utilized to perform track association, and then the extrinsic parameters were calculated, which were compared with the ground truth provided in the dataset. Each set of the CARRADA datasets contained 2–4 targets. The experimental results are given in Table 6, with an average rotation error (ARE) of 0.81° and an average translation error (ATE) of 0.0754 m, which are also shown in Figure 12.

Due to the influence of the outliers, the ARE and ATE of approach A were 1.5267° and 0.2597 m. The ARE and ATE of approach B were 3.7054° and 0.4540 m, which were caused by incorrect track pairs.

**Figure 13:**
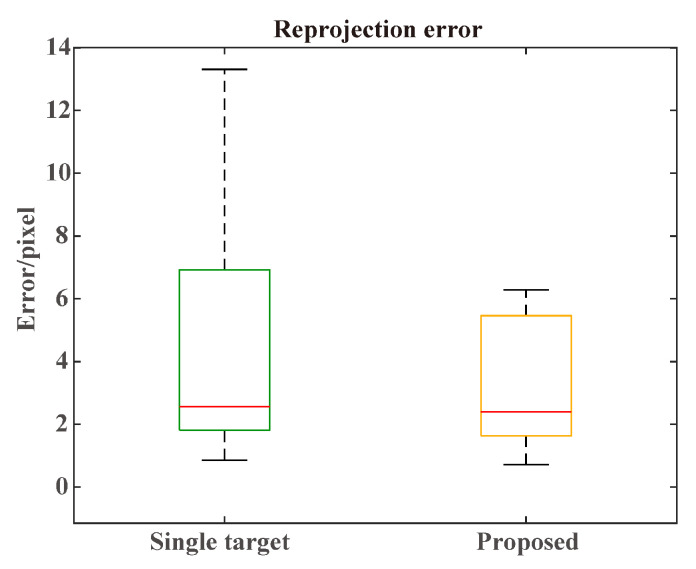
Reprojection error of the proposed algorithm and single-target calibration on the CARRADA dataset.

**Figure 14:**
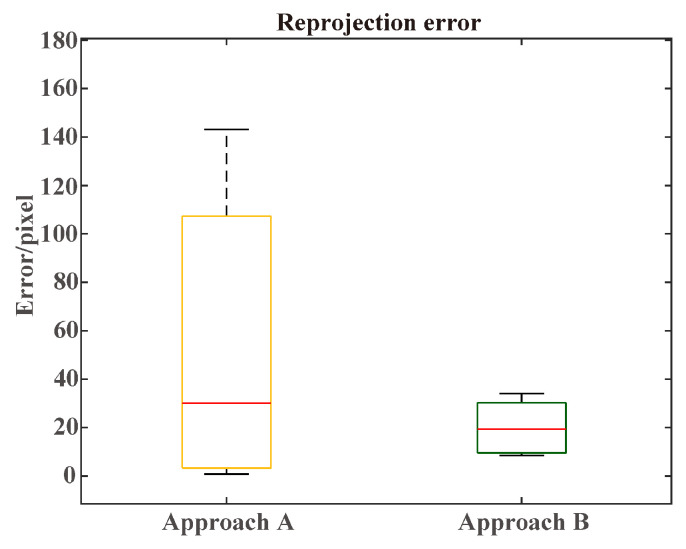
Reprojection error of approaches A and B on the CARRADA dataset.

**Figure 15:**
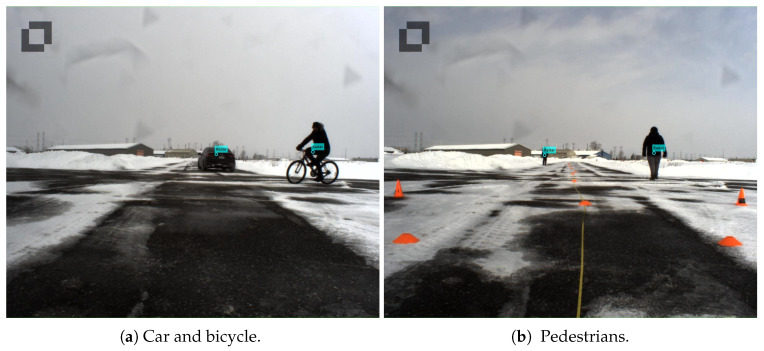
The scenario of the CARRADA dataset.

The radar detection projection in the image was compared between the extrinsic parameters obtained from multiple track pairs and a single track pair. The single track pair was selected by choosing the track pair with the minimum elements in the cost matrix M. As shown in Figure 13, applying multiple track pairs could effectively reduce the reprojection error by 1/3. The REs for the single target and multiple targets were 4.6979 and 3.1613 pixels. The calibration from multiple track pairs achieved the best performance, which proved the importance of accurate track association. The reprojection errors of the two comparison methods are shown in Figure 14. In certain scenarios, approach A achieved accurate track association, which led to a lower reprojection error. However, due to the track association error in other situations, the overall performance of approach A was worse than approach B. Figure 15 shows a visualization of the calibration results.

### 5.3. Robustness Test

To validate the robustness of the proposed algorithm, we conducted robustness tests by adding noise to real-world dataset. We selected the cubic polynomial fit of actual radar target tracks as the ground truth target track. In the experiments, four target tracks were considered, each had Gaussian white noise added in the *x* and *y* directions. The noise variance was set to 0.02:0.02:0.20 ($m^{2}$) and was equal in the *x* and *y* directions. The calibration errors were analyzed in terms of rotation error, translation error, and reprojection error.

**Figure 16:**
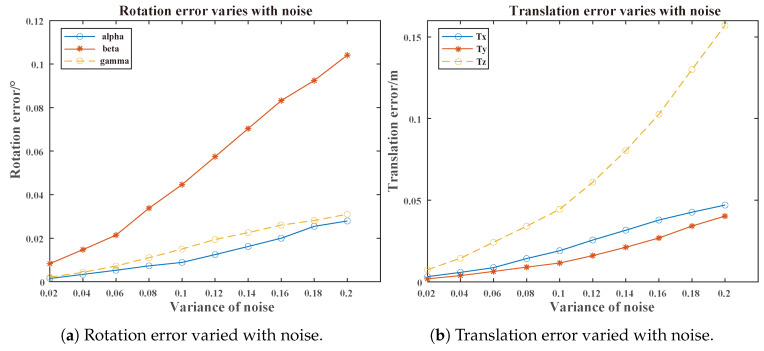
Rotation and translation error varied with noise.

**Figure 17:**
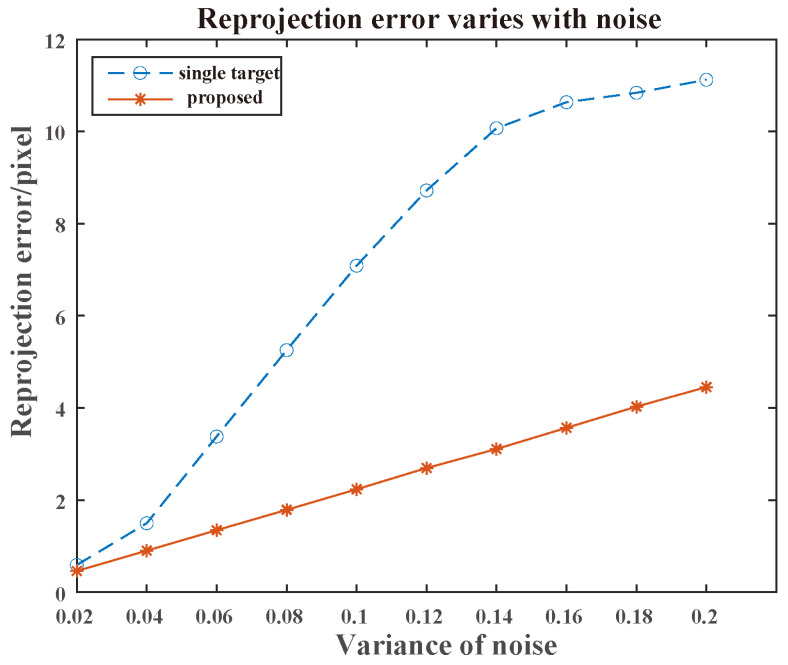
Reprojection error varied with noise.

1. Rotation and translation error: Figure 16 illustrates the variation in the calibration errors for rotation and translation as noise increased. Even under the maximum noise condition, the average rotation error remained below 0.1°, while the average translation error was below 0.1 m. As the noise variance increased, the variation trends of angles and translations in different directions exhibited slight differences, which may be attributed to the positions of the targets and the sensor within the scene.
2. Reprojection error: Figure 17 shows the variation in the calibration errors with increasing noise for the single-target track scenarios and multiple-target track scenarios (with a quantity of 4). Under the same noise condition, the calibration from multiple tracks (as proposed in our algorithm) achieved a lower reprojection error. Furthermore, it is vital to note that many calibration schemes relying on single-target scenarios fail when multiple targets are present.

## 6. Conclusions

In this paper, a novel algorithm for associating target tracks from heterogeneous sensors was proposed, and its capability to calibrate radar and camera systems was demonstrated. Unlike conventional methods that assume known extrinsic parameters between sensors, the challenging problem of associating tracks when they are unknown is addressed by our approach. Remarkably, explicit calibration objects or rough estimates of the extrinsic parameters are not required by our calibration method, ensuring precise calibration between radar and camera systems. Furthermore, specific calibration environments are not relied upon by the proposed approach, which solely leverages the information acquired by the radar and camera sensors.

Through outdoor experiments, the algorithm was validated, achieving an average reprojection error of 2.6649 pixels and a track association accuracy of 96.43%. Moreover, on the CARRADA dataset, an average reprojection error of 3.1613 pixels was attained by our algorithm. Compared to the extrinsic parameters obtained through manual calibration provided by the dataset, a significantly improved performance was exhibited by our algorithm, with an average rotation error of only 0.8141° and an average translation error of just 0.0754 m, demonstrating that the effect of our proposed algorithm was significantly better than the comparison methods, which also validated the effectiveness of the algorithm for targets such as vehicles and bicycles.

To further demonstrate the robustness of the algorithm, white Gaussian noise was added to the real-world target tracking data. The proposed algorithm consistently achieved an average rotation error below 0.1° and an average translation error below 0.1 m for the radar target tracks. These results highlight the accuracy and robustness of the proposed algorithm compared to the calibration scheme employing a single target.

In future work, we aim to apply this track association algorithm to other sensor systems, such as lidar and cameras. We may work on improving the computational complexity and optimizing the tracking algorithms. Additionally, we anticipate leveraging the proposed calibration algorithm to construct a robust sensor system capable of automatically correcting extrinsic parameters.

## Figures and Tables

**Table 1:** Evaluation of radar target tracking.

Parameters	Values
MAE in the x-direction	0.1112 m
MAE in the y-direction	0.1740 m
MAE	0.2411 m
time to generate point cloud per frame	0.7514 s
time to tracking per frame	0.8044 s

**Table 2:** Parameters of AWR1443 FWCW radar.

Parameters	Values
Start Frequency $f_{c}$	77 GHz
Number of frames $N_{f}$	256
Number of chirps in one frame $N_{c}$	64
Number of samples in one frame $N_{s}$	256
Sweep bandwidth *B*	301.75 MHz
Number of Tx	3
Number of Rx	4
Range resolution ΔR	0.497 m
Velocity resolution ΔV	0.038 m/s
Frame period	60 ms

**Table 3:** RE of different methods in outdoor experiment.

Method	RE	Type
Single target calibration	5.9168 pixels	Targetless
Proposed	2.6649 pixels	Targetless
A [19]	31.53 pixels	Targetless
B [20]	11.998 pixels	Targetless
C [33]	1.47 pixels	Target-based
D [11]	6.29 pixels	Target-based
E [12]	15.31 pixels	Target-based

**Table 4:** MA of different methods in outdoor experiment.

Method	MA
*M* (Proposed)	96.43%
$L_{\mathrm{self}}$	71.43%
$L_{\mathrm{valid}}$	64.29%
A [19]	35.7%
B [20]	−
C [33]	−

**Table 5:** Average time consumption of different methods in outdoor experiment.

Method	Time	Type
Proposed	177.7638 s	Targetless
A [19]	112.6054 s	Targetless
B [20]	109.2323 s	Targetless
C [33]	−	Target-based

**Table 6:** Experimental results with the CARRADA dataset.

Method	RE	ATE	ARE
A [19]	19.3691 pixels	0.2597 m	1.5267°
B [20]	53.8791 pixels	0.4540 m	3.7054°
Proposed	3.1613 pixels	0.0754 m	0.8141°

## Data Availability

The data presented in this study are openly available in the CARRADA repository available at https://github.com/valeoai/carrada_dataset, accessed on 30 January 2025.
